# Single Nucleotide Polymorphisms in MicroRNA Binding Sites: Implications in Colorectal Cancer

**DOI:** 10.1155/2014/547154

**Published:** 2014-12-24

**Authors:** Panchalee Bhaumik, Chandrasekhar Gopalakrishnan, Balu Kamaraj, Rituraj Purohit

**Affiliations:** School of Bio Sciences and Technology (SBST), Bioinformatics Division, Vellore Institute of Technology University, Vellore, Tamil Nadu 632014, India

## Abstract

Cancer is a complex genetic disorder, characterised by uncontrolled cell proliferation and caused by altered expression of oncogenes and tumour suppressor genes. When cell proliferation pertains to colon, it is called colorectal cancer. Most of colorectal cancer causing genes are potential targets for the miRNA (microRNA) that bind to 3′UTR (untranslated regions) of mRNA and inhibit translation. Mutations occurring in miRNA binding regions can alter the miRNA, mRNA combination, and can alter gene expression drastically. We hypothesized that 3′UTR mutation in miRNA binding site could alter the miRNA, mRNA interaction, thereby altering gene expression. Altered gene expression activity could promote tumorigenesis in colon. Therefore, we formulated a systematic in silico procedure that integrates data from various databases, followed rigorous selection criteria, and identified mutations that might alter the expression levels of cancer causing genes. Further we performed expression analysis to shed light on the potential tissues that might be affected by mutation, enrichment analysis to find the metabolic functions of the gene, and network analysis to highlight the important interactions of cancer causing genes with other genes to provide insight that complex network will be disturbed upon mutation. We provide in silico evidence for the effect of these mutations in colorectal cancer.

## 1. Background

Colorectal cancer refers to colon or rectal cancer and most of them are of glandular origin and hence can be classified as adenocarcinomas. It can also be called bowel cancer and is the third most common type of cancer in the world with 45 out of 100,000 people suffering from the same according to the National Institute of Cancer statistics as of 2013. These two types of cancers are significantly similar in their genomic mutations and besides that bear symptomatic semblance [1]. Colorectal cancer is characterized by tumors that form in the tissues of the colon or the rectum. Like tumors in general, these too are formed as a result of the abnormal and uncontrolled division of cells. The causes of colorectal cancer are mostly unknown, although it may be inherited or genetically unrelated [2].

A tumor in colon occurs mainly due to altered expression of two kinds of genes, proto-oncogenes and tumor suppressor gene. Proto-oncogenes are genes, which encode proteins that play a pivotal role in the colon tissue division. Increased expression of proto-oncogenes will result in an increased rate of cell divisions that may lead to cancer. Tumor suppressor genes, on the other hand, encode proteins that would arrest the proliferation of the cancer, by initiating cell apoptosis. Underexpression of tumor suppressor genes will not arrest the tumor; thereby it will help in cancer cell proliferation. Functionally, these genes show to be involved in a number of biological processes and molecular functions such as phosphorylation, regulation and modification of proteins, binding, and signaling. In this study, we have given yet another cause for altered gene expression of proto-oncogenes and tumor suppressor genes (through the mutation of the miRNA binding site of the genes) which may lead to tumorigenesis.

**Figure 1:**
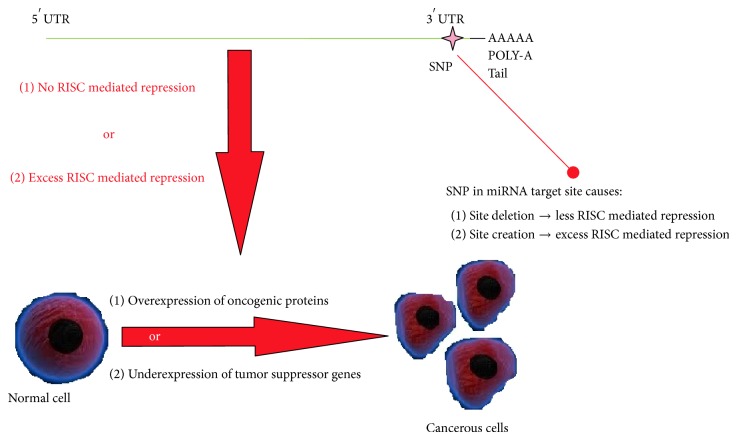
Concept diagram.

With disease genetics becoming an increasingly investigated field, SNP analysis is becoming an area that is being extensively looked into for a clearer picture of the root cause of a disease. SNPs (single nucleotide polymorphisms) are singular allele changes in genes that can cause erroneous gene translation or produce incorrect proteins. SNPs are more often found in the noncoding regions of the gene rather than the functional coding elements [3]. The coding regions of a gene usually cause changes in product protein conformation. In the case of mutations in noncoding regions, gene expressions are more likely to get affected [4]. We concentrated on studying the effects of the existence of SNPs on microRNA target sites available in mRNA. Many studies also implicate SNPs in microRNA networks, in the increased risk of cancer [5]. MicroRNAs (miRNAs) are approximately 22-nucleotide long RNAs that have proven important in regulatory functions of organisms [6]. They accomplish through mRNA target site cleavage or repression by disruption of translational initiation. Part of a miRNA is a 2–8-base pair long seed region in their 5′ end. The interaction happens between these seed regions and complementary “seed matches” on the target sites of the mRNAs [7]. Target site variants may sometimes cause a change in the binding specificity of miRNAs thus giving rise to improper binding and hence the leaky translation [8–11]. This it does by the involvement of the RNA induced silencing complex (RISC). RISC contains a site complementary to miRNA seed regions, which can be used to detect these miRNAs. Succeeding in this, it inhibits the translation of the detected gene by cleaving the RNA thereby reducing the expression and protein formation. However, SNP mutations can alter the expression of these genes by either creating or deleting binding sites. The creation of new binding sites can cause extensive RISC mediated repression whereas the loss of a binding site can lead to failure to attract RISC and hence allow uninhibited expression. Both results could cause cancer. Other than the creation of new binding sites, the SNPs may enhance, decrease, or completely disrupt the binding efficacy of miRNAs. Creation of binding sites leads to overexpression and a decrease in the same escorts to underexpression of a gene [12]. Due to the vast amount of literature available on the genomics of colorectal cancer, we could collect and analyze gene-related data to understand which genes could play a significant role in disease caused as a result of common genomic alterations. This project concentrated on 54 major colorectal cancer related genes and narrowed them down to 34 genes that had miRNA binding sites in their 3′ UTRs. These 34 were further filtered based on their capacity to create binding sites and increase their efficiency or delete them and decrease their efficiency. Genes such as BCL2 and MET have shown lesser tendencies to create sites than to have to delete them, hence making them less prone to RISC mediated degradation and more easily expressed. BCL2 has been shown to antagonize apoptotic cell death and MET has been known to lead metastatic properties of cells [13, 14]. Correspondingly, TP53 and SMAD2/3 have shown a proclivity to create sites making them more prone to repression. TP53 is known as a tumor suppressor and has been shown to be repressed in colorectal cancer and SMAD2/3 has been shown to be mutated in most cases of colorectal cancer and possibly most underexpressed [13, 15]. Thus, with the help of a systematic computational protocol of analysis and filtering we could isolate certain genes that can considerably alter and correct genetic functioning due to predicted mutations in their nucleotide sequences. Given the functions of the above-described genes, it can be revealed that the under- or overexpression is crucial to the manifestation of cancer. The analysis of microRNA target site SNPs corroborating these facts says that these SNPs also might have a determining role to play in the cause of disease. The overall concept of these studies was shown in Figure 1.

## 2. Materials and Methods

### 2.1. Dataset

To isolate the genes that played major roles in the manifestation of colorectal cancer, we surveyed literature from various research groups to form a comprehensive list [16–18]. The data were further refined and updated from online databases and websites such as the National Cancer Institute and the Atlas of Genetics and Cytogenetics in Oncology and Haematology database [19]. We found 54 genes, 16 of which were defined as major genes. These are essential genes which on their own are capable of causing disease on account of certain well recognized and common mutations that may occur in them [20].

### 2.2. Identification and Analysis of the miRNA Target Site SNPs

The server used to obtain the TS (target site) information was MirSNP (http://202.38.126.151/hmdd/mirsnp/search/). A gene list was uploaded via its batch gene upload option. The MAF (minor allele frequency) filter was kept on, which filtered an MAF >0.01 in at least one population of four [21]. The results were displayed in a tabular format and the miRNA and TS data we retrieved from it were sorted out into four categories:

 create: when a mutation tends to create a new mRNA binding site that another gene can bind to,
 break: when a mutation tends to destroy a binding site, thus causing repression of a gene,
 enhance: when a mutation enhances the binding efficiency between the miRNA and the TS,
 decrease: when a mutation decreases the binding efficiency between the miRNA and the TS.

A list was put together of the expression profiles of these genes which pointed out that they all fell under three different categories: some were strongly expressed; some showed weak expression; and others showed the proclivity to neither greater nor lesser expression.

### 2.3. Retrieving the EST Profiles of Our Genes

Expressed sequence tag (EST) profiles are retrieved from the NCBI UniGene online server (http://www.ncbi.nlm.nih.gov/UniGene/). These profiles are displayed as colored gray to black dots of different intensity of color under different heads or categories [22]. We made use of the “Breakdown by Body Sites” header and the “Breakdown by Health State” header. Two tables were created, one showing the approximate expression profile of the chosen gene in colorectal cancer and the other showing the estimated expression of the same genes in different tissues of the body. The intensity of a dot represents an estimate of the number of ESTs collected of that gene for that particular disease from the servers CDNA library sources. This helps infer the expression patterns of the genes.

### 2.4. Functional Annotation and Enrichment Analysis

For the analysis of enrichment of function, the WebGestalt server (http://bioinfo.vanderbilt.edu/webgestalt/) was used [23]. We uploaded our gene list in batch under the “hsapiens” organism option and the “gene symbol” id type option. We used the “hypergeometric” statistical method. The resultant functional analysis was tabulated.

### 2.5. Creating a Gene Network

We created gene networks for different combinations of genes using the GeneMania online software at http://www.genemania.org/ [24]. GeneMania gives us a graphic representation of a network in which our selected genes are related. The software uses a gradient optimization algorithm to relate the chosen genes according to their functional annotation data sources.

## 3. Results and Discussion

### 3.1. Target Site SNPs Identified

To screen out the genes of interest, we fed in a batch, a list of the HNGC gene symbols that we used, to the MirSNP server. We filtered our results with a minor allele frequency (MAF) >0.1%. From an initial list of 54 genes, we narrowed it down to 34, which had SNPs on their miRNA target sites, as seen in Table 1; the complete version of create or enhance and break or delete of SNP in the miRNA binding sites is shown in Tables S1 and S2 in Supplementary Material available online at http://dx.doi.org/10.1155/2014/547154, respectively.

**Figure 2:**
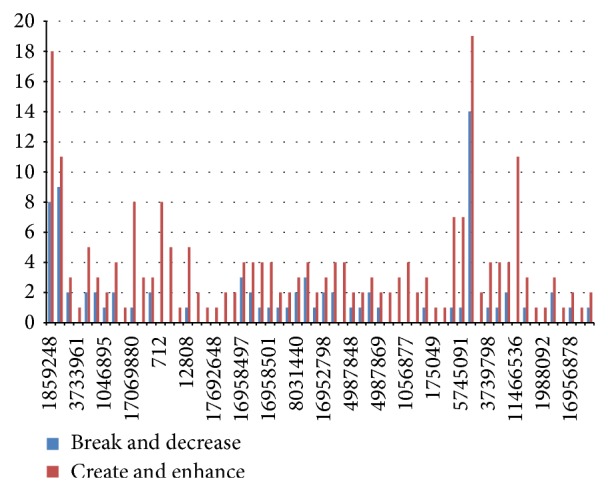
This chart shows the level of the increase in expression against its decrease. Here, the blue bars represent site breakage and decrease in binding efficiency of miRNAs and the red bars indicate the creation of sites and the increase in binding efficiency. The *x*-axis corresponds to the number of microRNAs an SNP can target and the *y*-axis shows the specific SNP id concerned. In this chart, we can see underexpression of genes because of the higher levels of creation and enhancement.

**Figure 3:**
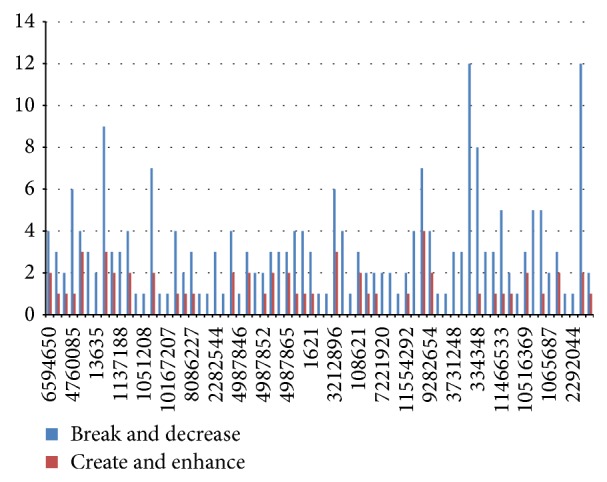
This chart like its previous counterpart shows overexpression. It does by higher levels of breakage and decrease (blue) of sites.

Thus, these genes were concluded to have altered gene expression patterns. Once we narrowed down the genes that actually can affect normal genetic functions, we needed to take a closer look at how they were doing the same by analyzing the data retrieved. The statistical representation of the number of SNPs in a microRNA target site that can break a binding site or reduce its efficiency against those that could create one or decrease its efficiency was shown in Figures 2 and 3. Hence, from Figure 2 we find a stronger incidence of creation and enhancement, which implies that the genes with these SNPs in their miRNA target sites have a tendency to be underexpressed. In Figure 3, on the other hand, chances of breakage or decrements are higher, pointing to the possible overexpression.

### 3.2. Analyzing the Mir-TS-SNP Results

The results retrieved from MirSNP were divided into four categories “create,” “break,” “enhance,” and “decrease.” We chose to compare the categories create and enhance against break and decrease. Create shows the number of new sites produced due to the SNP while enhance indicates that the SNP can cause an increase in the binding efficiency of the miRNA to the target site. Break shows the number of sites that were disrupted due to genetic alteration and decrease shows the tendency to decrease the miRNA binding efficiency. The tendency to create sites or enhance the binding efficiency would promote extensive RISC mediated translation inhibition or degradation and thus cause underexpression. On the other hand, breakage or disruption of the sites or the decrease in binding efficiency could block RISC mediated translation inhibition and hence give an excess of the protein product; that is, it would promote overexpression. On comparing these two categories, we found our results confirming the roles of many of the genes we worked with. While some are known tumor suppressors and oncogenes showed higher chances of under- and overexpression, respectively, still others pointed to being equally susceptible to both.

### 3.3. EST Profile Analysis

In order to find out the regions that might be affected upon mutation, we performed the expression analysis. This helps understand the extent to which these genes are expressed and thus play a part in colorectal cancer. UniGene contains a repository of expression profiles of diverse genes, each being displayed by white to black ellipses of modulating intensities shown in Table 2. The darker ellipse indicated the more expressed gene. In UniGene expression is measured under various categories of which we chose the “Breakdown by Body Sites” category (Table S3). This showed us a consistent expression of all our selected genes in the intestine. The colon and rectum are both parts of the large intestine and thus strong expression in these parts supports the possibility of these genes working as contributing factors to both colon and rectal cancer. To ascertain the above, we also retrieved results from the “Breakdown by Health State” category from which we retrieved the expression profiles of genes in colorectal cancer (Table 2). Table 2 gives the gene expression profile of genes at the time of colorectal cancer. If we compare the intensities of the genes from Table 2 (during colorectal cancer) with the intensities of genes for intestine (Table S3, normal states) we find that the genes chosen show varying levels of expression represented by the change in intensity (difference in shades). All of them show significant levels of varied expression, which relates them to colorectal cancer. Thus we propose that the mutation in miRNA binding site could be a reason for change in gene expression. However, varying intensities show some genes to be overexpressed like the MET proto-oncogene and others like the TP53 tumor suppressor gene to be underexpressed. These results corroborate with the experimentally proved nature of these genes.

### 3.4. Enrichment Analysis

To understand the functioning of our genes, we used the WebGestalt server (WEB-based GEne SeT AnaLysis Toolkit: http://bioinfo.vanderbilt.edu/webgestalt/). This server gave us a comprehensive list of all the biological and molecular processes that our selected genes were involved in. It provided an insight to readers on the metabolic functions that are likely to be affected upon the altered gene expressions. Among these, protein phosphorylation was one. Phosphorylation of proteins usually occurs to activate proteins to take part in cellular reactions. Most importantly though were the SMAD binding functions. SMAD binding helps mediate a signaling process involving the TGF beta superfamily, which ultimately affects cell proliferation and differentiation. The regulations of cellular and protein metabolic processes are also important functions that were highlighted. All these functions propose that an alteration in the expression of these genes could lead to abnormal functioning, proliferation, and migration of cells. A detailed look into the functioning of the above genes can be found in Table 3. Signaling functions can be pointed out to the WNT receptor signaling pathway. This pathway has three classifications which each had different functions of transcription, cytoskeleton structure, and calcium regulation in the cell.

### 3.5. Gene Network Information

**Figure 4:**
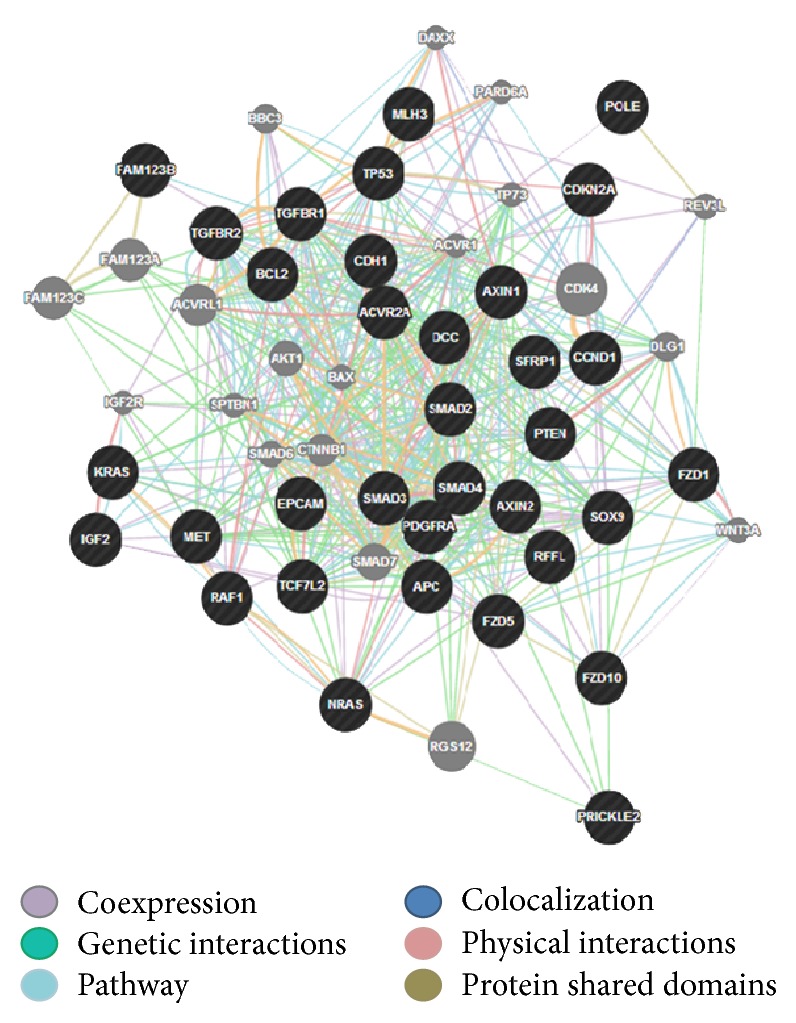
This is a representation of all 34 genes that were initially selected as major contributors to colorectal cancer and how they are related to each other in terms of pathways, colocalization, coexpression, shared protein domains, predicted genes, and physical and genetic interactions.

**Figure 5:**
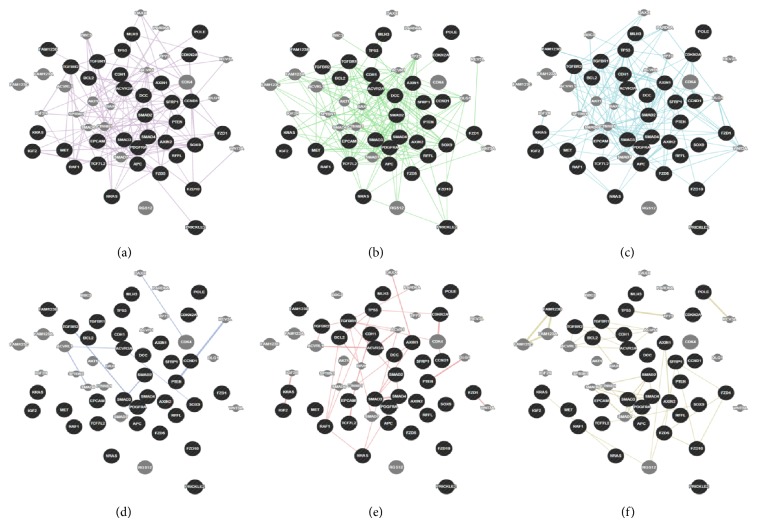
This figure is a representation of all the individual networks retrieved from GeneMania. From the top left to the bottom right, they display coexpression, genetic interactions, and pathways, coexpression, protein-protein interactions, and shared protein domains.

Once we were sure of the gene's role in colorectal cancer, our next step was to look for any existing links between our genes and other genes in a network (that are likely to be affected upon mutation and altered gene expression). Genetic interaction, pathway, and coexpression were factors that strongly linked a majority of our chosen genes as can be seen. In terms of physical interactions, TP53 seemed to be a hub of interactions as it linked itself to the SMAD genes (SMAD2/3/4) and TGFBR1 on one hand and also showed the relation to the MET proto-oncogene and BCL2 tumor promoter. Many other genes as well related indirectly to one another through predicted genes. STK11 seemed to have the majority of shared protein domains, thus linking these genes with it: TGFBR1/2, AVCR2A, AVCR1B, ERBB3, MET, and RAF1. MLH1 showed strong colocalization with genes MSH2 and STK11 as did SMAD2 with SMAD3. PCNA and REV3L are genes that were suggested by GeneMania and showed distinct linkages with quite a few query genes such as TP53, MSH6, POLD1, and PTEN, respectively. The various linkages have been elucidated in the results as displayed in Figures 4 and 5. We developed the significance of the linkages between over- and underexpressed genes by studying the pathways that they were involved in corresponding to their relationship with other genes. BCL2 and TP53 proved to be an excellent case of over- and underexpression of genes together promoting colorectal cancer. The BCL2 gene has proven in studies to be an apoptotic inhibitor [13]. The abnormal activation or overexpression of this gene causes the inhibition of apoptosis or programmed cell death. TP53, on the other hand, is a well-known tumor suppressor. It helps in suppression by promoting apoptosis of malignant cells or cells with damaged DNA. BCL2 and TP53 have been shown to work in relation to each other to inhibit apoptotic cell death. Their expression rates have been shown to be inversely related and are also common in colorectal cancer [25].

SMAD2/3 or RSMADs are a part of the SMAD family of genes, which play an important role in most types of cancer development. They are involved in the TGF beta signaling SMAD dependent pathway and help mediate transcription within the nucleus. It has been shown that members with the SMAD family help transmit signals from the cell surface located TGF beta superfamily in the nucleus [26]. Around 20 percent of all colorectal cancer cases show mutations in SMAD2 and higher frequency of occurrence of SMAD2/3 increases the possibility of seeing these genes as tumor suppressors and regulators of development [15]. In correspondence to these facts, our results also pointed out the underexpression of SMAD2/3 genes. The MET gene has been pointed out to be a potent tumor promoter in cases of cancer. However, it has also been widely associated with colorectal cancer and SLM (synchronous liver metastasis) [14]. Studies show MET as instrumental in providing a selective advantage for cell growth of neoplastic cells. Amplification of this gene has also been associated with lending metastatic properties of cells [27]. MET was shown to be expressed 50 times as much in colorectal cancer in any stage of progression [14].

Our results showed the overexpression of MET thus supporting the aforementioned facts. In the above represented gene networks, pathways can be traced out by following the light blue lines in the networks

The green lines represent genetic interactions. Genetic interactions happen when genes are related to each other by function and disturbing one gene would affect the other. These are indicated by green lines. Protein-protein interactions are marked in red lines. These reflect that the proteins belonging to the two connected genes have been indicated by studies, to interact. Gold lines represent data on shared domains. If the protein domains of two gene proteins are shared, we see this connecting line. The coexpression is denoted by lilac lines and marks genes that have shown similar levels of expression under the identical conditions of a study. Colocalization normally refers to genes that express their proteins in the same locations; these genes are shown to be connected by bluish gray lines. Lastly in colocalization, which generally refers to genes that express their proteins in the same locations, these genes are shown to be connected by bluish gray lines.

## 4. Conclusion

After a thorough screening of genes that play a determining role in colorectal cancer, we could isolate genes with SNPs in their mRNA target sites. This was followed by a reverse analysis of the number of created or disrupted sites and their binding efficiencies. A study of their expression profiles confirmed the roles of the chosen genes in colorectal cancer. This implicated such genes as SMAD2/3 and TP53 to be underexpressed and MET and BCL2 as overexpressed. An enrichment analysis helped us understand their functions among which were TGF beta signaling and regulation of phosphorylation of proteins, which proved to play an important role in certain biochemical pathways related to colorectal cancer. Connecting the dots using gene networks helped establish the roles of these genes in well laid down pathways and their ability to bestow properties that are crucial to apoptotic cell death, metastasis, and SMAD signaling in the TGF beta SMAD dependent pathway. BCL2, TP53, MET, and SMAD2/3 were all found to play regulatory roles in the above-mentioned areas and their altered expression proved to be complicit with the development of colorectal cancer. At every step, we reconfirmed the importance of microRNA target site SNPs in the prediction of colorectal carcinoma by verifying the results of the alteration in their expression levels due to excessive or insufficient RISC mediated translational inhibition. Concentrating future studies on miRNA target site SNPs could be beneficial in that they could provide information on gene expression profiles in disease and determine the role and importance of a gene for a disease. It is our belief that if these genes were studied more thoroughly they could be exploited for their therapeutic properties.

## Supplementary Material

Table S1: The mutations of category ‘‘create” would create new binding sites for miRNAs. This would make more miRNAs to target tumour suppressor genes gene. As a result, there would be lower expression of genes. The lower expression of tumour suppressor genes would cause aneuploidy (which may cause colorectal cancer). The mutations of category ‘‘enhance” would stabilise the miRNA–mRNA interactions that exist already in the cell. They also play a vital role in under expression of tumour suppressor genes.
Table S2: The mutations of category ‘‘break” would delete the miRNA binding sites in the oncogenes. Consequently, natural control of gene expression is lost; this would result in overexpression of genes. Overexpression of oncogenes genes would result in uncontrolled cell division (thereby cancer). The mutation in the category ‘‘decrease” will destabilise the miRNA–mRNA interaction. The miRNA–mRNA complex may or may not break. They play little role in regulation of gene expression.

## Figures and Tables

**Figure 6:**
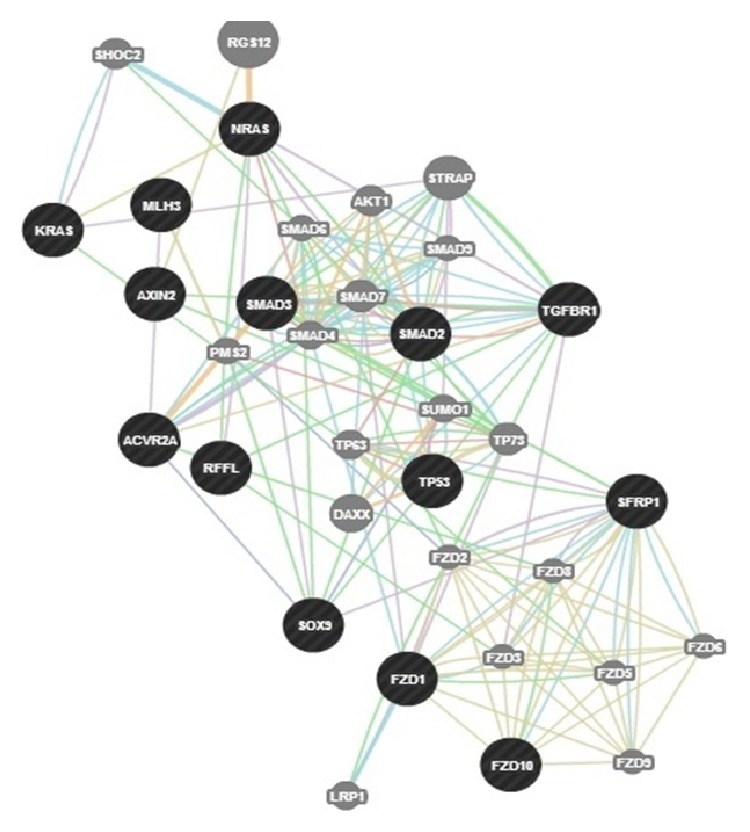
This network was derived from the GeneMania tool from genemania.org. It creates a network between the genes that were found to be underexpressed. The blue lines represent the various pathways connecting our genes of interest, which are in turn shaded in black. Gray spheres are genes that are not queried genes but are predicted to be related.

**Figure 7:**
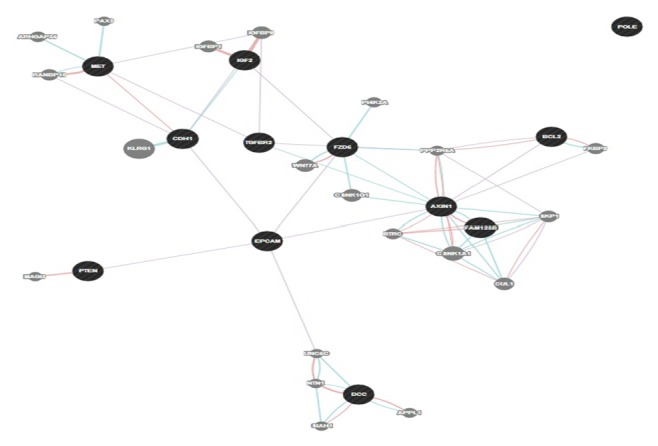
The network created for the overexpressed genes. Blue lines indicate pathways between spheres (Figures 6 and 7). Black spheres are query genes, and gray spheres show related or predicted genes not among the query genes.

**Table 1:** Genes with disrupted or reduced efficiency binding sites, suggesting underexpression.

Gene symbol	rsID	miRNA	Allele change
RFFL	1859248	6 microRNAs	A ⟹ G
		6 microRNAs	C ⟹ G
		2 microRNAs	C ⟹ T
		2 microRNAs	G ⟹ T
	3744358	2 microRNAs	G ⟹ T
		2 microRNAs	A ⟹ G
		2 microRNAs	C ⟹ G
		2 microRNAs	A ⟹ C
		hsa-miR-3143	A ⟹ T
		2 microRNAs	C ⟹ T
	12947086	4 microRNAs	A ⟹ G

APC	6594650	2 microRNAs	A ⟹ C
	3733961	hsa-miR-5686	A ⟹ G

FZD1	1052015	5 microRNAs	A ⟹ C
	3750145	3 microRNAs	A ⟹ G
	13403275	hsa-miR-4497	A ⟹ G

FZD10	1046890	2 microRNAs	C ⟹ T
	1046893	hsa-miR-4719	C ⟹ G
	1046895	2 microRNAs	A ⟹ G
	3741568	hsa-miR-4698	A ⟹ G
	4760085	hsa-miR-5683	C ⟹ G

PRICKLE2	14056	2 microRNAs	A ⟹ G
	26937	3 microRNAs	A ⟹ C
	26938	hsa-miR-552	G ⟹ T
	26939	hsa-miR-92b-5p	A ⟹ G
	27383	2 microRNAs	A ⟹ G
	153732	hsa-miR-4490	A ⟹ G
	17069879	hsa-miR-320e	A ⟹ G
		8 micrRNAs	C ⟹ G

SFRP1	1127379	3 microRNAs	A ⟹ G
	3242	hsa-miR-4789-3p	C ⟹ T

PDGFRA	7680422	hsa-miR-1256	A ⟹ C
	1565664	3 micriRNAs	G ⟹ T

KRAS	712	8 microRNAs	G ⟹ T
	9266	5 microRNAs	C ⟹ T
	12587	hsa-miR-506-5p	A ⟹ C
	13096	2 microRNAs	A ⟹ G
	1137282	hsa-miR-2681-5p	A ⟹ T
		hsa-miR-2681-5p	C ⟹ T
	7960917	hsa-miR-5700	C ⟹ T
	7973623	hsa-miR-4495	A ⟹ G

RAF1	12808	3 microRNAs	C ⟹ T
	5746246	hsa-miR-548an	C ⟹ T
	5746247	2 microRNAs	C ⟹ T

ACVR2A	2854464	hsa-miR-149-5p	A ⟹ G
	6734630	2 microRNAs	A ⟹ G
	11831802	hsa-miR-411-5p	C ⟹ T
	12993800	hsa-miR-5583-3p	C ⟹ T
	13430086	hsa-miR-876-5p	A ⟹ T
	17692648	hsa-miR-508-3p	A ⟹ C

SMAD2	1981	hsa-miR-100-3p	C ⟹ T
	1792671	2 microRNAs	A ⟹ G
	8085335	hsa-miR-3653	A ⟹ G
	8086227	hsa-miR-645	A ⟹ G
	8098413	2 microRNAs	C ⟹ T
	16958495	hsa-miR-3975	A ⟹ G
	16958497	4 microRNAs	A ⟹ T
	16958498	4 microRNAs	C ⟹ T
	16958499	4 microRNAs	A ⟹ C
	16958501	4 microRNAs	G ⟹ T
	16958509	2 microRNAs	C ⟹ T

SMAD3	1052488	2 microRNAs	C ⟹ T
	3743342	6 microRNAs	C ⟹ T
	8031440	3 microRNAs	A ⟹ G
	8031627	hsa-miR-596	A ⟹ G
	11556090	4 microRNAs	A ⟹ G
	12900401	2 microRNAs	C ⟹ T

SMAD4	4940037	2 microRNAs	A ⟹ G
	16952798	3 microRNAs	A ⟹ C

BCL2	1016860	4 microRNAs	A ⟹ G
	1564483	hsa-miR-4440	A ⟹ G
	3744937	hsa-miR-3911	C ⟹ T
	4987843	4 microRNAs	A ⟹ G
	4987847	2 microRNAs	A ⟹ G
	4987848	2 microRNAs	A ⟹ G
	4987850	2 microRNAs	A ⟹ G
	4987852	hsa-miR-1229	A ⟹ G
	4987853	2 microRNAs	A ⟹ G
	4987854	hsa-miR-497-3p	A ⟹ G
	4987855	2 microRNAs	A ⟹ G
	4987856	hsa-miR-3160-5p	A ⟹ G
	4987859	hsa-miR-3944-5p	A ⟹ G
	4987861	2 microRNAs	C ⟹ T
	4987865	2 microRNAs	A ⟹ G
	4987868	3 microRNAs	A ⟹ C
	4987869	2 microRNAs	A ⟹ C

AXIN1	394128	hsa-miR-3649	C ⟹ T
	393521	hsa-miR-653	G ⟹ T

MET	1621	hsa-miR-1284	A ⟹ G
	41739	2 microRNAs	A ⟹ G

AXIN2	7591	3 microRNAs	A ⟹ T

TCF7L2	1056877	4 microRNAs	C ⟹ T

CCND1	7177	hsa-miR-939	A ⟹ C
	7178	hsa-miR-4757-5p	A ⟹ G
	3212895	2 microRNAs	A ⟹ G
	3212896	3 microRNAs	G ⟹ T
	3212905	hsa-miR-155-3p	A ⟹ G
	3212908	3 microRNAs	C ⟹ T

MLH3	108621	2 microRNAs	C ⟹ T
	108622	3 microRNAs	A ⟹ G
	175049	hsa-miR-573	C ⟹ T

TP53	4968187	hsa-miR-3168	C ⟹ T
	17882252	hsa-miR-3615	A ⟹ G
	17879353	hsa-miR-4524b-3p	A ⟹ C

PTEN	701848	hsa-miR-3658	C ⟹ T

EPCAM	11554292	hsa-miR-466	A ⟹ T

POLE	14302	7 microRNAs	C ⟹ T
	5745091	7 microRNAs	A ⟹ G

CDH1	13689	hsa-miR-4435	A ⟹ C
		hsa-miR-4435	A ⟹ T
		hsa-miR-4435	C ⟹ T
		hsa-miR-4435	G ⟹ T
	9282654	2 microRNAs	G ⟹ T

CDKN2A	3088440	3 microRNAs	A ⟹ G
		4 microRNAs	C ⟹ G
		hsa-miR-1908	A ⟹ C
		3 microRNAs	A ⟹ T
		4 microRNAs	C ⟹ T
		4 microRNAs	G ⟹ T
	3731255	2 microRNAs	C ⟹ G
	6413463	hsa-miR-3683	A ⟹ T

TGFBR1	1590	2 microRNAs	A ⟹ C
	334349	2 microRNAs	A ⟹ G
	420549	hsa-miR-4753-3p	C ⟹ G
	3739798	4 microRNAs	C ⟹ T
	7850895	4 microRNAs	C ⟹ T
	10988732	hsa-miR-384	C ⟹ T

TGFBR2	11466531	hsa-miR-5708	C ⟹ G
	11466533	hsa-miR-4329	A ⟹ G
	11466534	4 microRNAs	A ⟹ G
	11466536	11 microRNAs	C ⟹ T
	11466537	hsa-miR-1193	A ⟹ T
	17026332	2 microRNAs	A ⟹ C

NRAS	14804	2 microRNAs	C ⟹ T
	1815675	hsa-miR-3143	G ⟹ T
	1988092	2 microRNAs	A ⟹ T
	2793257	3 microRNAs	C ⟹ G
	9724642	hsa-miR-1282	A ⟹ G
	10516369	2 microRNAs	A ⟹ T

IGF2	7873	hsa-miR-3191-3p	A ⟹ G

DCC	2270954	2 microRNAs	A ⟹ C
	12607853	2 microRNAs	C ⟹ T
	16956878	2 microRNAs	C ⟹ T

SOX9	1042667	hsa-miR-1181	A ⟹ C

FAM123B	3810701	2 microRNAs	A ⟹ C
	5964736	hsa-miR-450a-3p	C ⟹ T
	28653713	2 microRNAs	G ⟹ T

**Table 2:** Expression profiles of genes in colorectal cancer, retrieved from UniGene.

Gene	Expression in colorectal cancer
RFFL	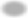
APC	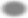
FZD1	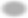
FZD5	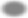
FZD10	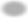
SFRP1	
KRAS	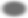
RAF1	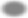
ACVR2A	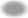
SMAD2	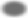
SMAD3	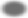
PDGFRA	
SMAD4	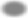
BCL2	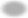
NRAS	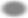
SOX9	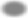
PRICKLE2	
AXIN1	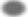
MET	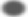
AXIN2	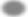
TCF7L2	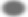
CCND1	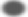
MLH3	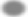
TP53	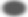
PTEN	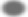
EPCAM	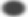
POLE	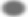
CDH1	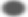
CDKN2A	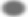
TGFBR1	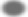
TGFBR2	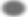
DCC	
IGF2	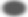
FAM123B	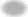

**Table 3:** Molecular functions and biological processes that the selected genes play a role in, along with their GO categories.

Biological processes, molecular functions, and GO Category	*R*	adjP
Regulation of phosphorylation GO:0042325	10.36	2.99*e* − 23
Regulation of protein phosphorylation GO:0001932	10.68	5.41*e* − 23
Protein phosphorylation GO:0006468	8.38	1.13*e* − 22
Phosphorylation GO:0016310	7.62	2.50*e* − 22
Regulation of signal transduction GO:000996	5.90	3.74*E* − 21
Positive regulation of protein metabolic process GO:0051247	9.57	4.11*e* − 21
Enzyme linked receptor protein signaling pathway GO:0007167	9.44	5.27*e* − 21
Positive regulation of cellular protein metabolic process GO:0032270	10.08	5.73*e* − 21
Regulation of protein modification GO:0031399	8.52	1.09*e* − 20
Regulation of signaling GO:0023051	5.31	1.35*e* − 20
SMAD binding GO:0046332	45.77	2.12*e* − 12
Beta-catenin binding GO:0008013	41.20	3.83*e* − 11
Protein kinase binding GO:0019901	11.98	3.83*e* − 11
I-SMAD binding GO:0070411	157.30	3.83*e* − 11
Mismatch repair complex GO:0032404	144.19	4.39*e* − 11
Enzyme binding GO:0019899	5.84	7.53*e* − 11
Kinase binding GO:0019900	10.84	9.22*e* − 11
Protein binding GO:0005515	1.93	1.21*e* − 10
Mismatched DNA binding GO:0030983	115.35	1.31*e* − 10
Transmembrane receptor protein kinase GO:0019199	30.90	1.99*e* − 10
