# Supplementary material for: Single Nucleotide Polymorphisms in MicroRNA Binding Sites: Implications in Colorectal Cancer
Source: ScientificWorldJournal. 2014 Dec 24;2014:547154. doi: 10.1155/2014/547154 (PMC4310495; doi:10.1155/2014/547154)

Table 1s: Complete details of the miRNA and the SNPs in their target sites.

	rsID	miRNA	Allele Change
RFFL	1859248	hsa-miR-3607-3p	A==>G
		hsa-miR-3607-3p	C==>G
		hsa-miR-4268	A==>G
		hsa-miR-4268	A==>T
		hsa-miR-4268	C==>G
		hsa-miR-4268	C==>T
		hsa-miR-4268	G==>T
		hsa-miR-4446-3p	A==>G
		hsa-miR-4446-3p	C==>G
		hsa-miR-4495	A==>T
		hsa-miR-4495	C==>T
		hsa-miR-4495	G==>T
		hsa-miR-4675	A==>G
		hsa-miR-4675	C==>G
		hsa-miR-4691-5p	A==>G
		hsa-miR-4691-5p	C==>G
		hsa-miR-4741	A==>G
		hsa-miR-4741	C==>G
	3744358	hsa-miR-3916	G==>T
		hsa-miR-1294	A==>G
		hsa-miR-1294	C==>G
		hsa-miR-141-3p	A==>C
		hsa-miR-200a-3p	A==>C
		hsa-miR-3143	A==>T
		hsa-miR-3143	C==>T
		hsa-miR-3143	G==>T
		hsa-miR-568	A==>G
		hsa-miR-568	C==>G
		hsa-miR-568	C==>T
	12947086	hsa-miR-1273g-5p	A==>G
		hsa-miR-4534	A==>G
		hsa-miR-4802-5p	A==>G
		hsa-miR-766-5p	A==>G
APC	6594650	hsa-miR-1322	A==>C
		hsa-miR-4296	A==>C
	3733961	hsa-miR-5686	A==>G
FZD1	1052015	hsa-miR-135a-5p	A==>C
		hsa-miR-135b-5p	A==>C

		hsa-miR-3607-5p	A==>C
		hsa-miR-4501	A==>C
		hsa-miR-4666a-5p	A==>C
	3750145	hsa-miR-4739	A==>G
		hsa-miR-4756-5p	A==>G
		hsa-miR-765	A==>G
	13403275	hsa-miR-4497	A==>G
FZD10	1046890	hsa-miR-4753-5p	C==>T
		hsa-miR-5007-5p	C==>T
	1046893	hsa-miR-4719	C==>G
	1046895	hsa-miR-3679-3p	A==>G
		hsa-miR-629-3p	A==>G
	3741568	hsa-miR-4698	A==>G
	4760085	hsa-miR-5683	C==>G
PRICKLE2	14056	hsa-miR-371b-5p	A==>G
		hsa-miR-373-5p	A==>G
	26937	hsa-miR-1292	A==>C
		hsa-miR-2392	A==>C
		hsa-miR-4448	A==>C
	26938	hsa-miR-552	G==>T
	26939	hsa-miR-92b-5p	A==>G
	27383	hsa-miR-4286	A==>G
		hsa-miR-4758-3p	A==>G
	153732	hsa-miR-4490	A==>G
	17069879	hsa-miR-320e	A==>G
		hsa-miR-3191-5p	C==>G
		hsa-miR-3192	C==>G
		hsa-miR-326	C==>G
		hsa-miR-330-5p	C==>G
		hsa-miR-4300	C==>G
		hsa-miR-4314	C==>G
		hsa-miR-5591-5p	C==>G
		hsa-miR-920	C==>G
SFRP1	1127379	hsa-miR-1266	A==>G
		hsa-miR-3127-5p	A==>G
		hsa-miR-3614-5p	A==>G
	3242	hsa-miR-4789-3p	C==>T
PDGFRA	7680422	hsa-miR-1256	A==>C
	1565664	hsa-miR-4452	G==>T
		hsa-miR-4520a-5p	G==>T
		hsa-miR-4520b-5p	G==>T
KRAS	712	hsa-miR-200b-3p	G==>T
		hsa-miR-200c-3p	G==>T

		hsa-miR-3125	G==>T
		hsa-miR-330-5p	G==>T
		hsa-miR-3916	G==>T
		hsa-miR-4646-5p	G==>T
		hsa-miR-514a-5p	G==>T
		hsa-miR-877-5p	G==>T
	9266	hsa-miR-181a-5p	C==>T
		hsa-miR-181b-5p	C==>T
		hsa-miR-181c-5p	C==>T
		hsa-miR-181d	C==>T
		hsa-miR-4262	C==>T
	12587	hsa-miR-506-5p	A==>C
	13096	hsa-miR-3177-5p	A==>G
		hsa-miR-4646-3p	A==>G
	1137282	hsa-miR-2681-5p	A==>T
		hsa-miR-2681-5p	C==>T
	7960917	hsa-miR-5700	C==>T
	7973623	hsa-miR-4495	A==>G
RAF1	12808	hsa-miR-4274	C==>T
		hsa-miR-4291	C==>T
		hsa-miR-4750	C==>T
	5746246	hsa-miR-548an	C==>T
	5746247	hsa-miR-1269a	C==>T
		hsa-miR-1269b	C==>T
ACVR2A	2854464	hsa-miR-149-5p	A==>G
	6734630	hsa-miR-124-3p	A==>G
		hsa-miR-506-3p	A==>G
	11831802	hsa-miR-411-5p	C==>T
	12993800	hsa-miR-5583-3p	C==>T
	13430086	hsa-miR-876-5p	A==>T
	17692648	hsa-miR-508-3p	A==>C
SMAD2	1981	hsa-miR-100-3p	C==>T
	1792671	hsa-miR-302b-5p	A==>G
		hsa-miR-302d-5p	A==>G
	8085335	hsa-miR-3653	A==>G
	8086227	hsa-miR-645	A==>G
	8098413	hsa-miR-3171	C==>T
		hsa-miR-32-3p	C==>T
	16958495	hsa-miR-3975	A==>G
	16958497	hsa-miR-4264	A==>T
		hsa-miR-4711-5p	A==>T
		hsa-miR-622	A==>T
		hsa-miR-767-3p	A==>T

	16958498	hsa-miR-181b-5p	C==>T
		hsa-miR-181d	C==>T
		hsa-miR-3688-3p	C==>T
		hsa-miR-5003-5p	C==>T
	16958499	hsa-miR-10b-3p	A==>C
		hsa-miR-4495	A==>C
		hsa-miR-513a-3p	A==>C
		hsa-miR-513c-3p	A==>C
	16958501	hsa-let-7a-3p	G==>T
		hsa-let-7b-3p	G==>T
		hsa-let-7f-1-3p	G==>T
		hsa-miR-5011-5p	G==>T
	16958509	hsa-miR-224-3p	C==>T
		hsa-miR-522-3p	C==>T
SMAD3	1052488	hsa-miR-20a-3p	C==>T
		hsa-miR-544a	C==>T
	3743342	hsa-miR-3609	C==>T
		hsa-miR-518a-5p	C==>T
		hsa-miR-520g	C==>T
		hsa-miR-520h	C==>T
		hsa-miR-527	C==>T
		hsa-miR-548ah-5p	C==>T
	8031440	hsa-miR-490-3p	A==>G
		hsa-miR-619	A==>G
		hsa-miR-676-5p	A==>G
	8031627	hsa-miR-596	A==>G
	11556090	hsa-miR-188-3p	A==>G
		hsa-miR-212-3p	A==>G
		hsa-miR-4433-5p	A==>G
		hsa-miR-5588-3p	A==>G
	12900401	hsa-miR-3611	C==>T
		hsa-miR-935	C==>T
SMAD4	4940037	hsa-miR-3686	A==>G
		hsa-miR-4766-5p	A==>G
	16952798	hsa-miR-4775	A==>C
		hsa-miR-4775	A==>C
		hsa-miR-590-3p	A==>C
BCL2	1016860	hsa-miR-27a-5p	A==>G
		hsa-miR-3127-5p	A==>G
		hsa-miR-3158-3p	A==>G
		hsa-miR-4720-3p	A==>G
	1564483	hsa-miR-4440	A==>G
	3744937	hsa-miR-3911	C==>T

	4987843	hsa-miR-1250	A==>G
		hsa-miR-449b-3p	A==>G
		hsa-miR-4746-3p	A==>G
		hsa-miR-636	A==>G
	4987847	hsa-miR-338-3p	A==>G
		hsa-miR-4310	A==>G
	4987848	hsa-miR-1234	A==>G
		hsa-miR-4269	A==>G
	4987850	hsa-miR-3922-5p	A==>G
		hsa-miR-4753-5p	A==>G
	4987852	hsa-miR-1229	A==>G
	4987853	hsa-miR-1298	A==>G
		hsa-miR-4686	A==>G
	4987854	hsa-miR-497-3p	A==>G
	4987855	hsa-miR-3943	A==>G
		hsa-miR-4779	A==>G
	4987856	hsa-miR-3160-5p	A==>G
	4987859	hsa-miR-3944-5p	A==>G
	4987861	hsa-miR-4722-3p	C==>T
		hsa-miR-4762-5p	C==>T
	4987865	hsa-miR-4729	A==>G
		hsa-miR-548a-3p	A==>G
	4987868	hsa-miR-1276	A==>C
		hsa-miR-548l	A==>C
		hsa-miR-548n	A==>C
	4987869	hsa-miR-3688-3p	A==>C
		hsa-miR-3978	A==>C
AXIN1	394128	hsa-miR-3649	C==>T
	393521	hsa-miR-653	G==>T
MET	1621	hsa-miR-1284	A==>G
	41739	hsa-miR-335-3p	A==>G
		hsa-miR-644b-3p	A==>G
AXIN2	7591	hsa-miR-548d-3p	A==>T
		hsa-miR-548h-3p	A==>T
		hsa-miR-548z	A==>T
TCF7L2	1056877	hsa-miR-330-3p	C==>T
		hsa-miR-548aa	C==>T
		hsa-miR-548ap-3p	C==>T
		hsa-miR-548t-3p	C==>T
CCND1	7177	hsa-miR-939	A==>C
	7178	hsa-miR-4757-5p	A==>G
	3212895	hsa-miR-4753-3p	A==>G
		hsa-miR-942	A==>G

	3212896	hsa-miR-3132	G==>T
		hsa-miR-4650-3p	G==>T
		hsa-miR-570-3p	G==>T
	3212905	hsa-miR-155-3p	A==>G
	3212908	hsa-miR-214-3p	C==>T
		hsa-miR-3619-5p	C==>T
		hsa-miR-761	C==>T
MLH3	108621	hsa-miR-3190-5p	C==>T
		hsa-miR-338-3p	C==>T
	108622	hsa-miR-4307	A==>G
		hsa-miR-548ai	A==>G
		hsa-miR-570-5p	A==>G
	175049	hsa-miR-573	C==>T
TP53	4968187	hsa-miR-3168	C==>T
	17882252	hsa-miR-3615	A==>G
	17879353	hsa-miR-4524b-3p	A==>C
PTEN	701848	hsa-miR-3658	C==>T
EPCAM	11554292	hsa-miR-466	A==>T
POLE	14302	hsa-miR-1343	C==>T
		hsa-miR-145-3p	C==>T
		hsa-miR-2110	C==>T
		hsa-miR-4667-5p	C==>T
		hsa-miR-4700-5p	C==>T
		hsa-miR-4782-5p	C==>T
		hsa-miR-5706	C==>T
	5745091	hsa-miR-1200	A==>G
		hsa-miR-3660	A==>G
		hsa-miR-4478	A==>G
		hsa-miR-4526	A==>G
		hsa-miR-4649-3p	A==>G
		hsa-miR-485-5p	A==>G
		hsa-miR-544b	A==>G
CDH1	13689	hsa-miR-4435	A==>C
		hsa-miR-4435	A==>T
		hsa-miR-4435	C==>T
		hsa-miR-4435	G==>T
	9282654	hsa-miR-4786-3p	G==>T
		hsa-miR-583	G==>T
CDKN2A	3088440	hsa-miR-1291	A==>G
		hsa-miR-1291	C==>G
		hsa-miR-1908	A==>C
		hsa-miR-1908	A==>T
		hsa-miR-1908	C==>T

		hsa-miR-1908	G==>T
		hsa-miR-328	A==>G
		hsa-miR-328	C==>G
		hsa-miR-548au-3p	C==>T
		hsa-miR-548au-3p	G==>T
		hsa-miR-663a	C==>G
		hsa-miR-663b	A==>G
		hsa-miR-663b	C==>G
		hsa-miR-744-3p	A==>T
		hsa-miR-744-3p	C==>T
		hsa-miR-744-3p	G==>T
		hsa-miR-874	A==>T
		hsa-miR-874	C==>T
		hsa-miR-874	G==>T
	3731255	hsa-miR-125b-5p	C==>G
		hsa-miR-138-1-3p	C==>G
	6413463	hsa-miR-3683	A==>T
TGFBR1	1590	hsa-miR-4286	A==>C
		hsa-miR-4758-3p	A==>C
	334349	hsa-miR-3612	A==>G
		hsa-miR-650	A==>G
	420549	hsa-miR-4753-3p	C==>G
	3739798	hsa-miR-297	C==>T
		hsa-miR-548ag	C==>T
		hsa-miR-548ai	C==>T
		hsa-miR-570-5p	C==>T
	7850895	hsa-miR-374a-5p	C==>T
		hsa-miR-374b-5p	C==>T
		hsa-miR-5692b	C==>T
		hsa-miR-5692c	C==>T
	10988732	hsa-miR-384	C==>T
TGFBR2	11466531	hsa-miR-5708	C==>G
	11466533	hsa-miR-4329	A==>G
	11466534	hsa-miR-2114-5p	A==>G
		hsa-miR-3183	A==>G
		hsa-miR-4723-3p	A==>G
		hsa-miR-642a-5p	A==>G
	11466536	hsa-miR-130a-3p	C==>T
		hsa-miR-130b-3p	C==>T
		hsa-miR-139-5p	C==>T
		hsa-miR-148a-3p	C==>T
		hsa-miR-148b-3p	C==>T
		hsa-miR-152	C==>T

		hsa-miR-301a-3p	C==>T
		hsa-miR-301b	C==>T
		hsa-miR-3666	C==>T
		hsa-miR-4295	C==>T
		hsa-miR-454-3p	C==>T
	11466537	hsa-miR-1193	A==>T
	17026332	hsa-miR-29a-5p	A==>C
		hsa-miR-4762-3p	A==>C
NRAS	14804	hsa-miR-5007-3p	C==>T
		hsa-miR-944	C==>T
	1815675	hsa-miR-3143	G==>T
	1988092	hsa-miR-4328	A==>T
		hsa-miR-4477b	A==>T
	2793257	hsa-miR-2110	C==>G
		hsa-miR-4261	C==>G
		hsa-miR-450a-3p	C==>G
	9724642	hsa-miR-1282	A==>G
	10516369	hsa-miR-1324	A==>T
		hsa-miR-589-3p	A==>T
IGF2	7873	hsa-miR-3191-3p	A==>G
DCC	2270954	hsa-miR-4759	A==>C
		hsa-miR-5190	A==>C
	12607853	hsa-miR-4704-5p	C==>T
		hsa-miR-590-3p	C==>T
	16956878	hsa-miR-128	C==>T
		hsa-miR-3681-3p	C==>T
SOX9	1042667	hsa-miR-1181	A==>C
FAM123B	3810701	hsa-miR-1973	A==>C
		hsa-miR-4536-3p	A==>C
	5964736	hsa-miR-450a-3p	C==>T
	28653713	hsa-miR-579	G==>T
		hsa-miR-644b-3p	G==>T

Table 2s: Complete table showing details of the miRNA and the SNPs with in their target sites. These suggest overexpression due to site breakage or decreased binding efficiency.

Gene Symbol	rs ID	miRNAs	Allele Change
RFFL	3744358	hsa-miR-1252	G==>T
		hsa-miR-1294	G==>T
		hsa-miR-141-3p	C==>G
		hsa-miR-141-3p	C==>T
		hsa-miR-1294	G==>T
		hsa-miR-141-3p	C==>G
		hsa-miR-141-3p	C==>T
		hsa-miR-200a-3p	C==>G
		hsa-miR-200a-3p	C==>T
	1859248	hsa-miR-296-3p	A==>C
		hsa-miR-296-3p	A==>G
		hsa-miR-296-3p	A==>T
		hsa-miR-3607-3p	G==>T
		hsa-miR-4446-3p	G==>T
		hsa-miR-4675	G==>T
		hsa-miR-4691-5p	G==>T
		hsa-miR-4741	G==>T
	3744359	hsa-miR-568	A==>C
		hsa-miR-568	A==>T

		hsa-miR-568	G==>T
	12947086	hsa-miR-629-5p	A==>G
APC	6875894	hsa-miR-135a-5p	C==>T
		hsa-miR-135b-5p	C==>T
		hsa-miR-3671	C==>T
		hsa-miR-5583-3p	C==>T
FZD1	1052015	hsa-miR-514a-3p	A==>C
		hsa-miR-514b-3p	A==>C
	3731568	hsa-miR-3613-3p	A==>C
	3750145	hsa-miR-219-2-3p	A==>G
		hsa-miR-766-5p	A==>G
	13403275	hsa-miR-4768-3p	A==>G
		hsa-miR-576-5p	A==>G
FZD5	13403275	hsa-miR-665	A==>G
FZD10	1046890	hsa-miR-4294	C==>T
		hsa-miR-4743	C==>T
	1046893	hsa-miR-455-3p	C==>G
	1046895	hsa-miR-1236	A==>G
	3741568	hsa-miR-4766-5p	A==>G
		hsa-miR-488-3p	A==>G
	4760085	hsa-miR-1321	C==>G
		hsa-miR-149-3p	C==>G
		hsa-miR-3162-5p	C==>G

		hsa-miR-4728-5p	C==>G
		hsa-miR-4739	C==>G
		hsa-miR-4756-5p	C==>G
PRIKCLE2	14056	hsa-miR-3688-5p	A==>G
		hsa-miR-616-5p	A==>G
	26937	hsa-miR-4426	A==>C
		hsa-miR-4477b	A==>C
		hsa-miR-4647	A==>C
		hsa-miR-4662b	A==>C
	26939	hsa-miR-4314	A==>G
	27383	hsa-miR-3916	A==>G
	12494731	hsa-miR-4534	G==>T
		hsa-miR-4802-5p	G==>T
		hsa-miR-766-5p	G==>T
	17069879	hsa-miR-582-3p	A==>G
	17069880	hsa-miR-216b	C==>G
SFRP1	3242	hsa-miR-3662	C==>T
	13635	hsa-miR-126-5p	A==>G
		hsa-miR-944	A==>G
PDGFRA	1565664	hsa-miR-1273g-5p	G==>T
		hsa-miR-3074-5p	G==>T
	7680422	hsa-miR-1256	C==>G
		hsa-miR-20a-3p	A==>C

		hsa-miR-20a-3p	A==>G
		hsa-miR-33a-5p	A==>G
		hsa-miR-33b-5p	A==>C
		hsa-miR-33b-5p	A==>G
KRAS	13096	hsa-let-7g-3p	A==>G
		hsa-miR-2355-3p	A==>G
		hsa-miR-676-3p	A==>G
	1137188	hsa-miR-1290	C==>T
		hsa-miR-541-5p	C==>T
		hsa-miR-511	C==>T
		hsa-miR-1248	A==>C
		hsa-miR-1248	A==>T
		hsa-miR-4724-3p	A==>C
		hsa-miR-4724-3p	A==>T
	7960917	hsa-miR-3912	C==>T
	7973450	hsa-miR-302c-5p	A==>G
RAF1	12808	hsa-miR-5007-5p	C==>T
	1051208	hsa-miR-4436b-5p	C==>T
	5746246	hsa-miR-4685-5p	C==>T
		hsa-miR-370	C==>T
		hsa-miR-378f	C==>T
		hsa-miR-378h	C==>T
		hsa-miR-4498	C==>T

		hsa-miR-5001-5p	C==>T
		hsa-miR-5002-3p	C==>T
		hsa-miR-762	C==>T
ACVR2A	2854464	hsa-miR-5580-5p	A==>G
	4664229	hsa-miR-4771	C==>T
	10167207	hsa-miR-362-5p	A==>C
	12993800	hsa-miR-4298	C==>T
		hsa-miR-4645-3p	C==>T
		hsa-miR-1302	C==>T
		hsa-miR-545-5p	C==>T
SMAD2	1981	hsa-miR-4766-5p	C==>T
		hsa-miR-488-3p	C==>T
	1792666	hsa-miR-3145-3p	A==>T
		hsa-miR-5695	A==>T
	8085335	hsa-miR-3133	A==>G
	8086227	hsa-miR-151a-3p	A==>G
		hsa-miR-4463	A==>G
		hsa-miR-5583-3p	A==>G
	16958497	hsa-miR-4700-3p	A==>T
		hsa-miR-4738-3p	A==>T
		hsa-miR-4772-3p	A==>T
	16958498	hsa-miR-181a-5p	C==>T
		hsa-miR-543	C==>T

	16958499	hsa-miR-205-3p	A==>C
	16958501	hsa-miR-4638-5p	G==>T
	16958509	hsa-miR-553	C==>T
	16958520	hsa-miR-2053	A==>G
SMAD3	1052488	hsa-miR-3682-5p	C==>T
	3743342	hsa-miR-2276	C==>T
		hsa-miR-4761-5p	C==>T
	3743343	hsa-miR-4679	C==>T
	8025774	hsa-miR-1249	C==>T
		hsa-miR-5687	C==>T
	8031440	hsa-miR-1299	A==>G
		hsa-miR-3065-5p	A==>G
	8031627	hsa-miR-3691-3p	A==>G
	11556090	hsa-miR-132-3p	A==>G
		hsa-miR-155-3p	A==>G
		hsa-miR-1910	A==>G
	12900401	hsa-miR-409-5p	C==>T
		hsa-miR-4289	C==>T
SMAD4	2282544	hsa-miR-186-3p	C==>G
		hsa-miR-330-3p	C==>G
		hsa-miR-4646-3p	C==>G
	3819122	hsa-miR-4517	G==>T
	4940037	hsa-miR-186-5p	A==>G

	16952798	hsa-miR-4775	A==>C
		hsa-miR-590-3p	A==>C
BCL2	1016860	hsa-miR-4789-5p	A==>G
		hsa-miR-629-5p	A==>G
	1564483	hsa-miR-296-3p	A==>G
		hsa-miR-4474-3p	A==>G
		hsa-miR-3653	A==>G
		hsa-miR-4666a-5p	A==>G
	4987846	hsa-miR-4761-3p	A==>G
	4987847	hsa-miR-16-1-3p	A==>G
		hsa-miR-4676-5p	A==>G
		hsa-miR-575	A==>G
	4987848	hsa-miR-4690-5p	A==>G
	4987850	hsa-miR-455-5p	A==>G
		hsa-miR-4717-5p	A==>G
	4987851	hsa-miR-3162-3p	A==>G
		hsa-miR-642b-5p	A==>G
	4987852	hsa-miR-1285-5p	A==>G
		hsa-miR-27a-5p	A==>G
	4987853	hsa-miR-4776-3p	A==>G
		hsa-miR-579	A==>G
		hsa-miR-644b-3p	A==>G
	4987854	hsa-miR-3974	A==>G

	4987855	hsa-miR-4779	A==>G
	4987856	hsa-miR-122-5p	A==>G
	4987858	hsa-miR-199a-5p	C==>T
		hsa-miR-2467-3p	C==>T
		hsa-miR-3678-3p	C==>T
	4987859	hsa-miR-143-5p	A==>G
	4987861	hsa-miR-4722-3p	C==>T
		hsa-miR-4762-5p	C==>T
	4987865	hsa-miR-217	A==>G
		hsa-miR-448	A==>G
		hsa-miR-548e	A==>G
	4987867	hsa-miR-4641	A==>G
	4987868	hsa-miR-548l	A==>C
		hsa-miR-548n	A==>C
	4987869	hsa-miR-4495	A==>C
AXIN1	393521	hsa-miR-320e	G==>T
		hsa-miR-3940-5p	G==>T
		hsa-miR-4507	G==>T
	394128	hsa-miR-1913	C==>T
		hsa-miR-324-3p	C==>T
		hsa-miR-4286	C==>T
		hsa-miR-4758-3p	C==>T
	393521	hsa-miR-5589-5p	G==>T

MET	1621	hsa-miR-199a-3p	A==>G
		hsa-miR-199b-3p	A==>G
		hsa-miR-3129-5p	A==>G
	41738	hsa-miR-4666a-5p	A==>G
TCF7L2	3793883	hsa-miR-296-3p	C==>G
CCND1	7177	hsa-miR-4697-3p	A==>C
	7178	hsa-miR-3941	A==>G
	678653	hsa-miR-602	C==>G
	3212896	hsa-miR-197-5p	G==>T
		hsa-miR-338-3p	G==>T
		hsa-miR-548aa	G==>T
		hsa-miR-548ap-3p	G==>T
		hsa-miR-548t-3p	G==>T
		hsa-miR-634	G==>T
	3212897	hsa-miR-125b-2-3p	G==>T
		hsa-miR-4327	G==>T
		hsa-miR-4731-3p	G==>T
		hsa-miR-4801	G==>T
	3212901	hsa-miR-2278	A==>C
	3212908	hsa-miR-342-5p	C==>T
		hsa-miR-4664-5p	C==>T
		hsa-miR-4781-5p	C==>T
MLH3	108621	hsa-miR-193a-3p	C==>T

		hsa-miR-193a-3p	C==>T
		hsa-miR-4435	C==>T
		hsa-miR-3145-3p	A==>G
TP53	17879353	hsa-miR-323b-5p	A==>C
		hsa-miR-377-5p	A==>C
	17882252	hsa-miR-4635	A==>G
PTEN	701848	hsa-miR-1304-5p	C==>T
		hsa-miR-5008-5p	C==>T
	7221920	hsa-miR-4650-3p	A==>G
		hsa-miR-4727-5p	A==>G
	11202607	hsa-miR-548at-5p	C==>T
		hsa-miR-5590-3p	C==>T
EPCAM	1421	hsa-miR-1277-5p	A==>G
	11554292	hsa-miR-379-3p	A==>T
		hsa-miR-411-3p	A==>T
	14302	hsa-miR-4532	C==>T
POLE		hsa-miR-1182	A==>G
		hsa-miR-125b-1-3p	A==>G
		hsa-miR-4782-5p	A==>G
		hsa-miR-5706	A==>G
	5745091	hsa-miR-3929	A==>G
CDH1	13689	hsa-miR-3117-5p	A==>C
		hsa-miR-3117-5p	A==>G

		hsa-miR-3117-5p	A==>T
		hsa-miR-4435	C==>G
		hsa-miR-4637	A==>C
		hsa-miR-4637	A==>G
		hsa-miR-4637	A==>T
	9282654	hsa-miR-129-1-3p	G==>T
		hsa-miR-129-2-3p	G==>T
		hsa-miR-671-5p	G==>T
		hsa-miR-938	G==>T
	13339481	hsa-miR-551b-5p	C==>T
CDKN2A	11515	hsa-miR-4502	C==>G
	3088440	hsa-miR-1291	G==>T
		hsa-miR-1908	C==>G
		hsa-miR-328	G==>T
		hsa-miR-486-3p	A==>C
		hsa-miR-486-3p	A==>G
		hsa-miR-486-3p	A==>T
		hsa-miR-548au-3p	A==>C
		hsa-miR-548au-3p	A==>G
		hsa-miR-548au-3p	A==>T
		hsa-miR-663a	A==>C
		hsa-miR-663a	A==>G
		hsa-miR-663a	A==>T

		hsa-miR-663a	G==>T
		hsa-miR-663b	G==>T
	3731249	hsa-miR-132-5p	A==>G
		hsa-miR-4642	A==>G
		hsa-miR-659-5p	A==>G
	6413463	hsa-miR-1306-3p	A==>T
	34011899	hsa-miR-1307-3p	A==>C
		hsa-miR-3669	A==>C
		hsa-miR-4268	A==>C
TGFBR1	868	hsa-let-7a-5p	A==>G
		hsa-let-7b-5p	A==>G
		hsa-let-7c	A==>G
		hsa-let-7d-5p	A==>G
		hsa-let-7e-5p	A==>G
		hsa-let-7f-5p	A==>G
		hsa-let-7g-5p	A==>G
		hsa-let-7i-5p	A==>G
		hsa-miR-1294	A==>G
		hsa-miR-3927	A==>G
		hsa-miR-4500	A==>G
		hsa-miR-98	A==>G
	334348	hsa-miR-591	A==>G
		hsa-miR-1275	A==>G
		hsa-miR-4447	A==>G

		hsa-miR-4472	A==>G
		hsa-miR-4665-5p	A==>G
		hsa-miR-4723-5p	A==>G
		hsa-miR-5698	A==>G
	420549	hsa-miR-511	C==>G
	3739798	hsa-miR-4666b	C==>T
	7850895	hsa-miR-2054	C==>T
	10988732	hsa-miR-5581-5p	C==>T
TGFBR2	6550008	hsa-miR-103a-3p	A==>G
		hsa-miR-107	A==>G
		hsa-miR-4310	A==>G
	11466531	hsa-miR-4419a	C==>G
		hsa-miR-4510	C==>G
		hsa-miR-5192	C==>G
	11466533	hsa-miR-204-5p	A==>G
		hsa-miR-211-5p	A==>G
		hsa-miR-4446-5p	A==>G
		hsa-miR-4755-5p	A==>G
		hsa-miR-5006-3p	A==>G
	11466534	hsa-miR-1910	A==>G
		hsa-miR-942	A==>G
	11466537	hsa-miR-3940-5p	A==>T
		hsa-miR-4507	A==>T

	17026332	hsa-miR-2116-5p	A==>C
		hsa-miR-4677-5p	A==>C
NRAS	14804	hsa-miR-218-5p	C==>T
	2793257	hsa-miR-3153	C==>G
		hsa-miR-4311	C==>G
	9724644	hsa-miR-181a-2-3p	A==>G
	10516369	hsa-miR-101-3p	A==>T
		hsa-miR-3614-3p	A==>T
		hsa-miR-4645-3p	A==>T
IGF2	2585	hsa-miR-28-5p	C==>T
		hsa-miR-3139	C==>T
		hsa-miR-3160-3p	C==>T
		hsa-miR-4682	C==>T
		hsa-miR-708-5p	C==>T
	7873	hsa-miR-185-3p	A==>G
		hsa-miR-4492	A==>G
		hsa-miR-4498	A==>G
		hsa-miR-5001-5p	A==>G
		hsa-miR-762	A==>G
	1065687	hsa-miR-4735-5p	A==>G
		hsa-miR-4775	A==>G
	3802971	hsa-miR-4711-5p	C==>T
DCC	2270954	hsa-miR-17-3p	A==>C

		hsa-miR-4504	A==>C	
		hsa-miR-4641	A==>C	
	2292043	hsa-miR-3689d	C==>T	
	2292044	hsa-miR-561-3p	C==>G	
	12607853	hsa-miR-141-3p	C==>T	
		hsa-miR-200a-3p	C==>T	
		hsa-miR-3654	C==>T	
		hsa-miR-378a-3p	C==>T	
		hsa-miR-378b	C==>T	
		hsa-miR-378c	C==>T	
		hsa-miR-378d	C==>T	
		hsa-miR-378e	C==>T	break
		hsa-miR-378f	C==>T	break
		hsa-miR-378h	C==>T	break
		hsa-miR-378i	C==>T	break
		hsa-miR-422a	C==>T	break
		hsa-miR-4775	C==>T	decrease
	16956878	hsa-miR-4666a-5p	C==>T	decrease
FAM123B	3810701	hsa-miR-148a-3p	A==>C	break
		hsa-miR-148b-3p	A==>C	break
		hsa-miR-152	A==>C	break
		hsa-miR-2682-5p	A==>C	break
		hsa-miR-34a-5p	A==>C	break
		hsa-miR-34b-5p	A==>C	break
		hsa-miR-34c-5p	A==>C	break
		hsa-miR-449a	A==>C	break
		hsa-miR-449b-5p	A==>C	break
		hsa-miR-449c-5p	A==>C	break
		hsa-miR-4514	A==>C	break
		hsa-miR-4692	A==>C	break

5964736	hsa-miR-2110	C==>T	decrease
	hsa-miR-4462	C==>T	break
2865371<br>3	hsa-miR-223-3p	G==>T	break

Table 3: Expression profiles of selected genes in different body tissues, retrieved from Unigene.

TISSUE/GENE	RFFL	APC	FZD1	FZD5	FZD10	SFRP1	RAF1	KRAS	PDGFRA	ACVR2A	SMAD2	SMAD3
ADIPOSE		●				●	●		●		●	
ADRENAL GLAND							●				●	
ASCITES			●	●			●	●			●	●
BLADDER		●	●					●			●	
BLOOD	●	●	●				●	●	●	●	●	●
BONE		●	●	●		●	●	●	●		●	
BONE MARROW	●						●	●	●		●	●
BRAIN	●	●	●	●		●	●	●	●	●	●	
CERVIX		●			●	●	●	●			●	
CONNECTIVE TISSUE	●	●	●	●	●	●	●	●	●	●	●	●
EAR	●	●	●			●	●	●	●		●	
EMBROYONIC TISSUE	●	●	●	●	●	●	●	●	●	●	●	●
ESOPHAGUS							●					●
EYE	●	●	●	●		●	●	●	●	●	●	●
HEART	●	●	●			●	●	●	●	●	●	●
INTESTINE	●	●	●	●	●	●	●	●	●	●	●	●
KIDNEY	●	●	●	●		●	●	●	●	●	●	●
LARYNX		●	●	●			●	●			●	

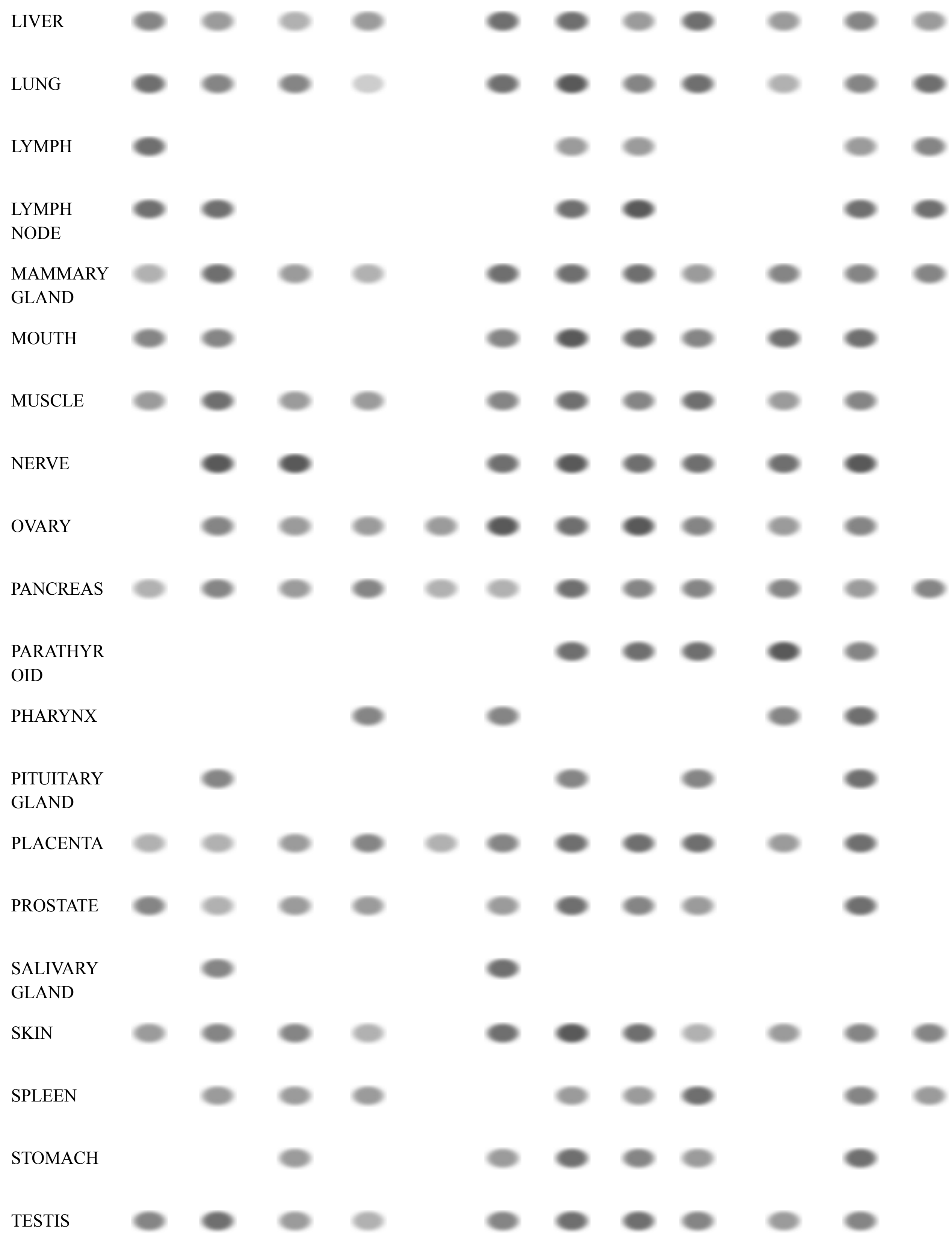
LIVER
LUNG
LYMPH
LYMPH NODE
MAMMARY GLAND
MOUTH
MUSCLE
NERVE
OVARY
PANCREAS
PARATHYR OID
PHARYNX
PITUITARY GLAND
PLACENTA
PROSTATE
SALIVARY GLAND
SKIN
SPLEEN
STOMACH
TESTIS

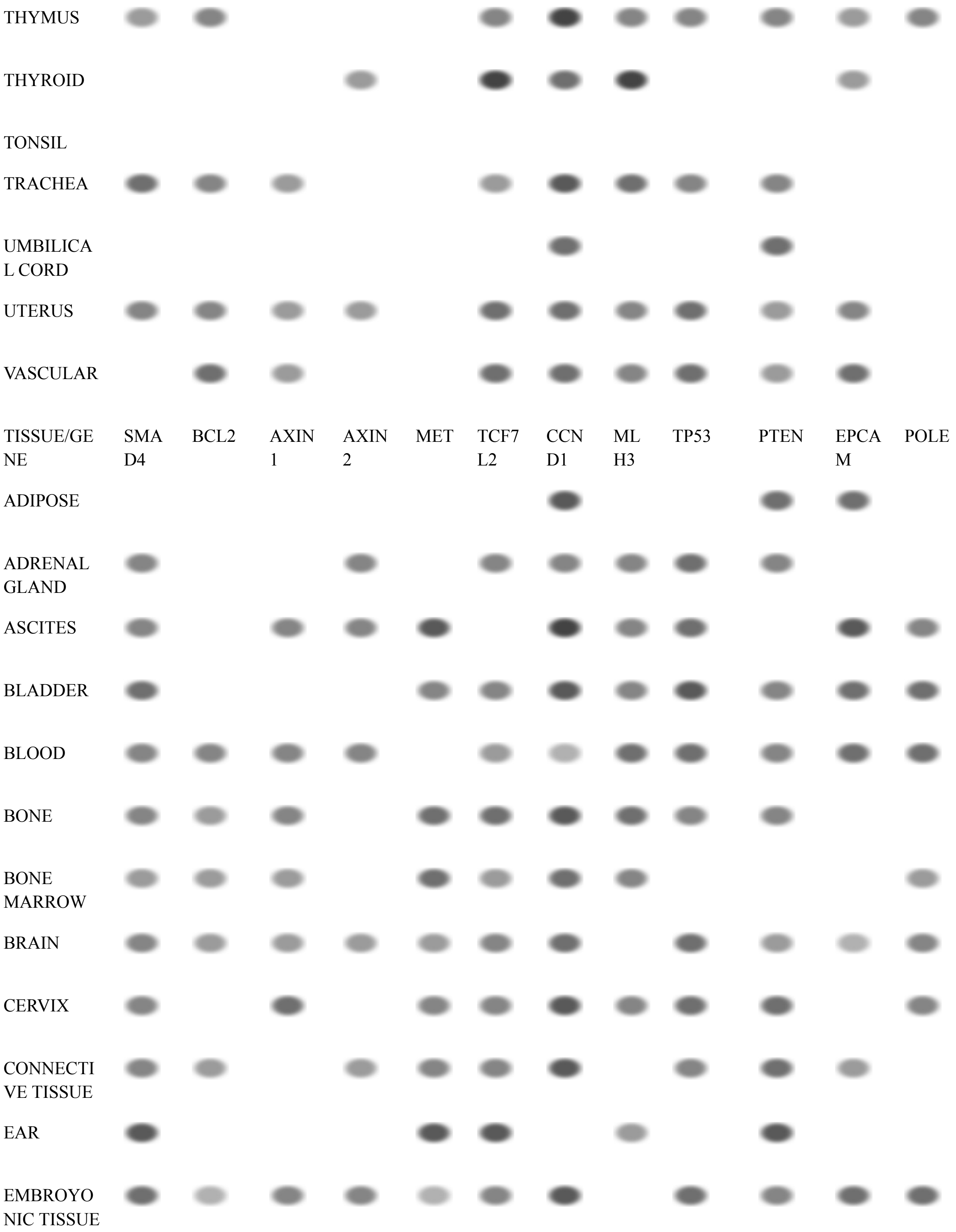

THYMUS
THYROID
TONSIL
TRACHEA
UMBILICAL CORD
UTERUS
VASCULAR
TISSUE/GENE
SMAD4
BCL2
AXIN1
AXIN2
MET
TCF7L2
CCND1
MLH3
TP53
PTEN
EPCAM
POLE
ADIPOSE
ADRENAL GLAND
ASCITES
BLADDER
BLOOD
BONE
BONE MARROW
BRAIN
CERVIX
CONNECTIVE TISSUE
EAR
EMBROYONIC TISSUE

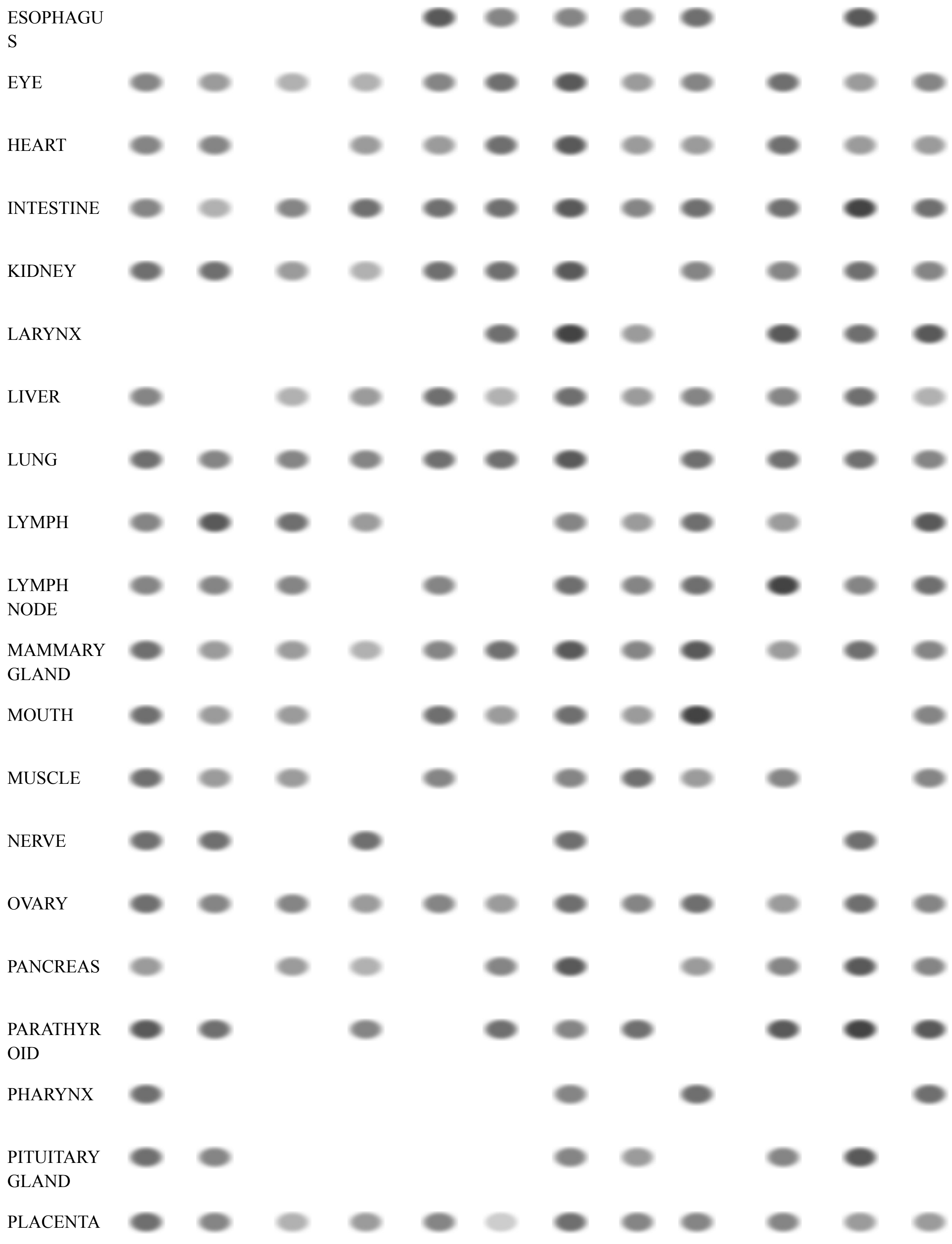

ESOPHAGUS
EYE
HEART
INTESTINE
KIDNEY
LARYNX
LIVER
LUNG
LYMPH
LYMPH NODE
MAMMARY GLAND
MOUTH
MUSCLE
NERVE
OVARY
PANCREAS
PARATHYROID
PHARYNX
PITUITARY GLAND
PLACENTA

PROSTATE	●	●	●	●	●	●	●		●	●	●	●
SALIVARY GLAND	●		●		●			●	●			
SKIN	●	●	●		●	●	●		●	●		●
SPLEEN	●		●		●	●		●	●	●		●
STOMACH	●	●	●		●	●	●	●	●	●	●	●
TESTIS	●	●	●	●	●	●	●	●	●	●	●	●
THYMUS	●	●		●	●	●	●		●	●		●
THYROID	●	●	●	●	●	●	●	●	●	●	●	●
TONSIL	●		●							●		
TRACHEA	●				●	●	●	●	●		●	●
UMBILICAL CORD								●		●		
UTERUS	●	●	●	●	●	●	●	●	●	●	●	●
VASCULAR	●	●			●	●	●		●	●	●	

TISSUE/GENE	CDH1	CDKN2A	TGFBR1	TGFBR2	NRAS	IGF2	DCC	SOX9		PRICKLE2
ADIPOSE				●		●				
ADRENAL GLAND			●	●	●	●				
ASCITES		●	●	●				●		
BLADDER	●			●	●				●	
BLOOD	●	●	●	●	●	●				

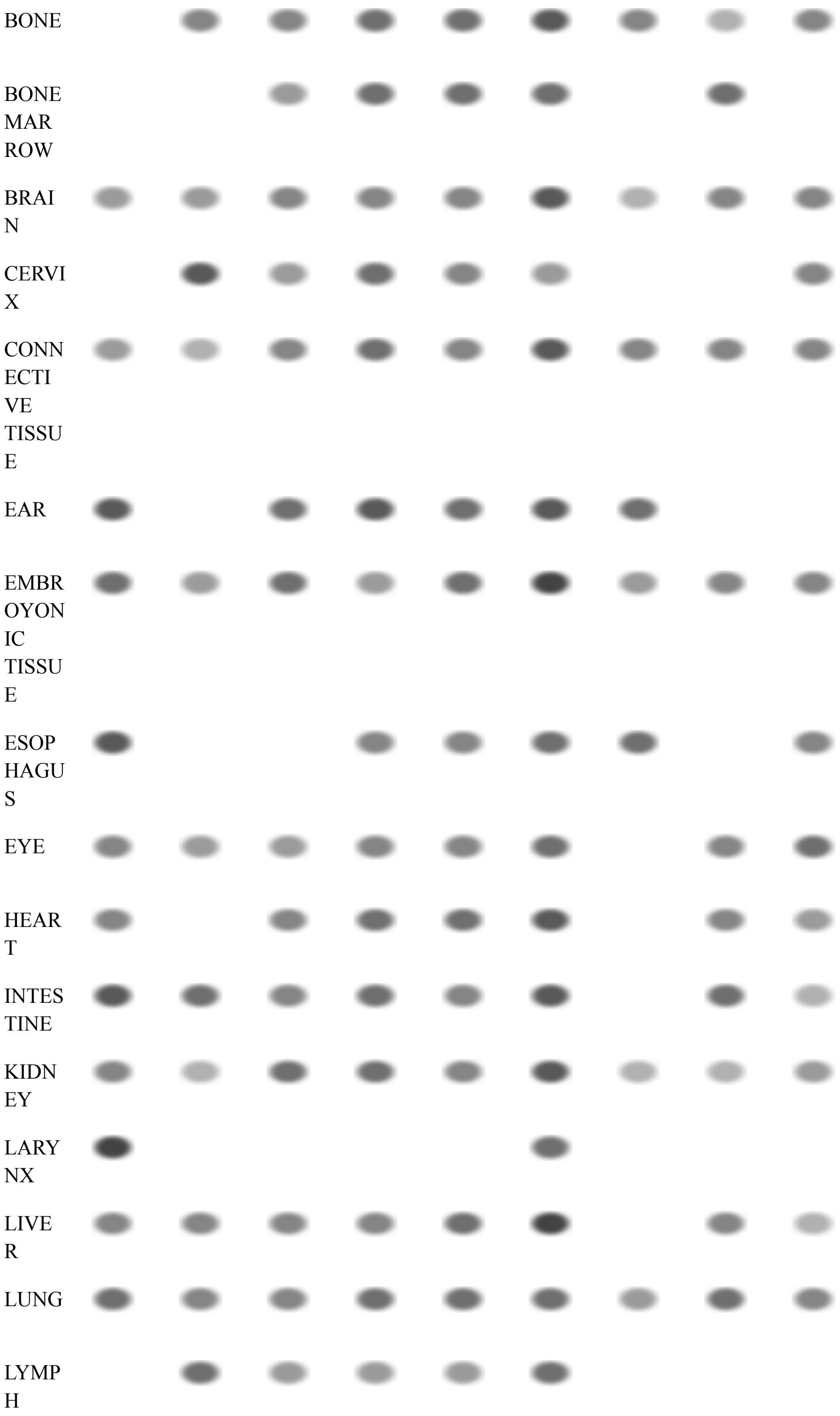
BONE
BONE MARROW
BRAIN
CERVIX
CONNECTIVE TISSUE
EAR
EMBROYONIC TISSUE
ESOPHAGUS
EYE
HEART
INTESTINE
KIDNEY
LARYNX
LIVER
LUNG
LYMPH

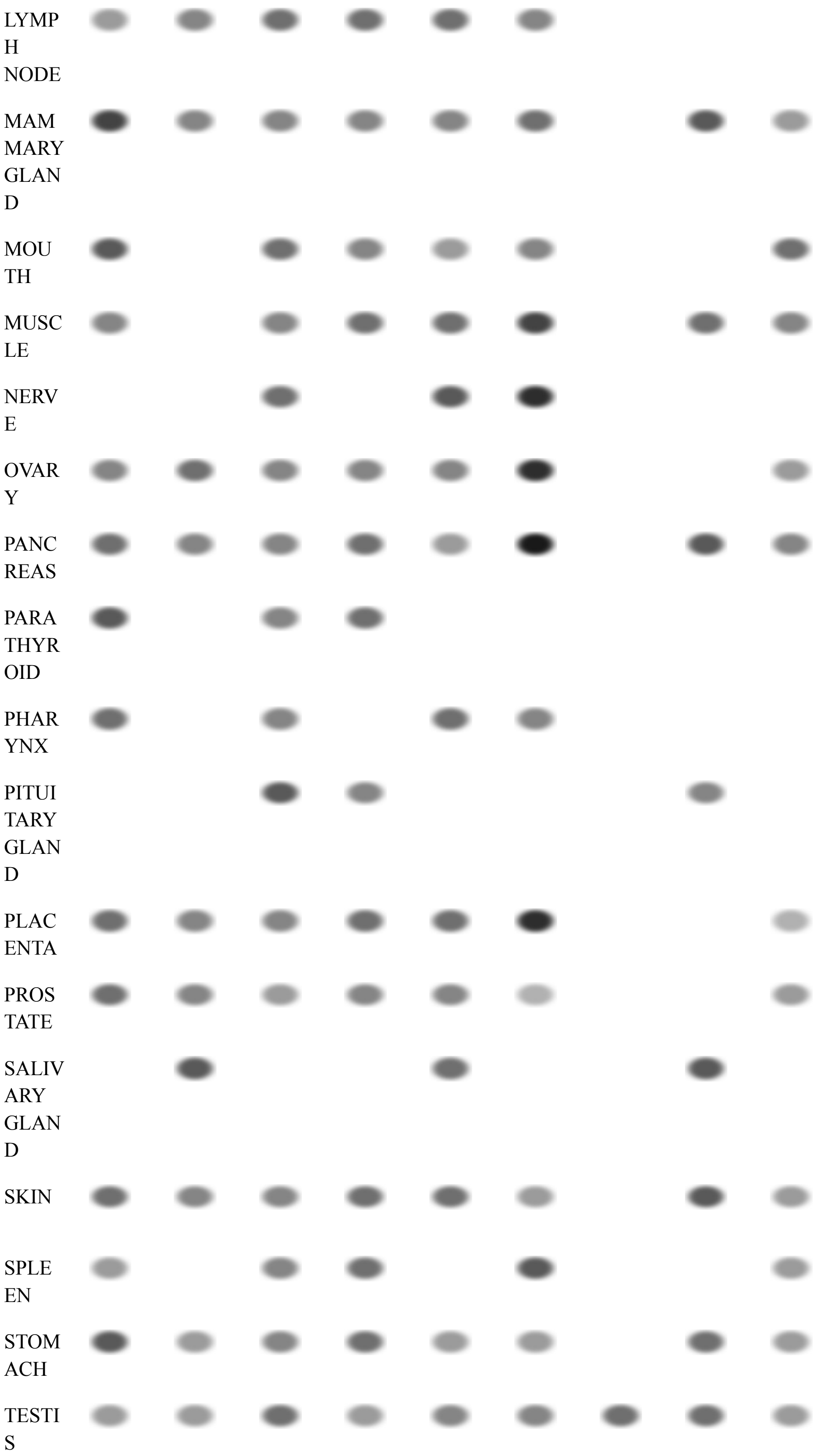
LYMPH NODE
MAMMARY GLAND
MOUTH
MUSCLE
NERVE
OVARY
PANCREAS
PARATHYROID
PHARYNX
PITUITARY GLAND
PLACENTA
PROSTATE
SALIVARY GLAND
SKIN
SPLEEN
STOMACH
TESTIS

THYMUS

THYROID

TONSIL

TRACHEA

UMBILICAL CORD

UTERUS

VASCULAR

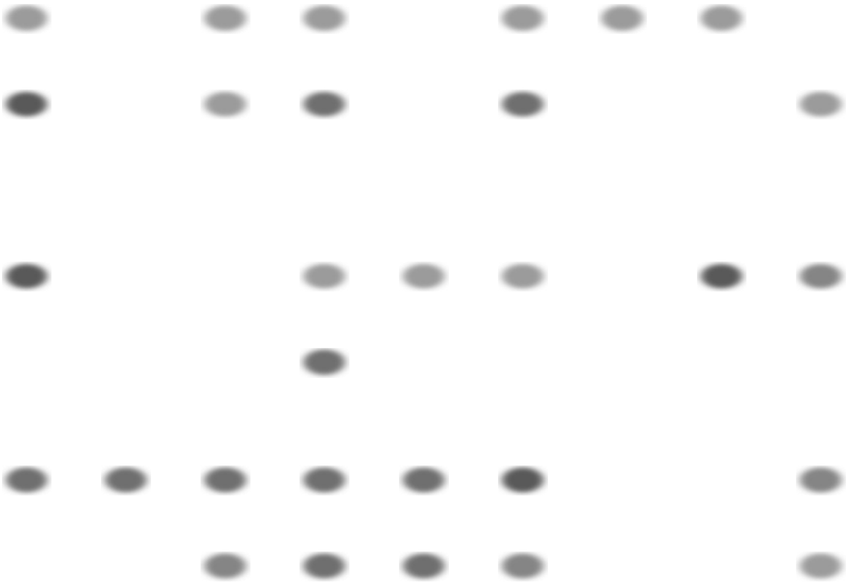

Supplement: Supplementary file 1 — Table S1: The mutations of category ‘‘create” would create new binding sites for miRNAs. This would make more miRNAs to target tumour suppressor genes gene. As a result, there would be lower expression of genes. The lower expression of tumour suppressor genes would cause aneuploidy (which may cause colorectal cancer). The mutations of category ‘‘enhance” would stabilise the miRNA–mRNA interactions that exist already in the cell. They also play a vital role in under expression of tumour suppressor genes. Table S2: The mutations of category ‘‘break” would delete the miRNA binding sites in the oncogenes. Consequently, natural control of gene expression is lost; this would result in overexpression of genes. Overexpression of oncogenes genes would result in uncontrolled cell division (thereby cancer). The mutation in the category ‘‘decrease” will destabilise the miRNA–mRNA interaction. The miRNA–mRNA complex may or may not break. They play little role in regulation of gene expression. [file 547154.f1.pdf]
